# Supplementary material for: Microporous Hydroxyapatite-Based Ceramics Alter the Physiology of Endothelial Cells through Physical and Chemical Cues
Source: J Funct Biomater. 2023 Sep 5;14(9):460. doi: 10.3390/jfb14090460 (PMC10531673; doi:10.3390/jfb14090460)
Supplement: Supplementary file 1 [file jfb-14-00460-s001.zip › jfb-2542512-supplementary.pdf]

Journal of
Functional
Biomaterials

MDPI

Supplementary Materials

Microporous Hydroxyapatite-Based Ceramics Alter the Physiology of Endothelial Cells through Physical and Chemical Cues

Julie Usseglio †, Adeline Dumur †, Esther Pagès, Émeline Renaudie, Alice Abélanet, Joël Brie, Éric Champion and Amandine Magnaudeix *

Institut de Recherche sur les Céramiques, UMR 7315, CNRS, Université de Limoges, F-87000 Limoges, France; julie.usseglio-grosso@unilim.fr (J.U.); adeline.dumur@etu.unilim.fr (A.D.); esther.pages@unilim.fr (E.P.); emeline.renaudie@unilim.fr (É.R.); alice.grenier-abelanet@unilim.fr (A.A.); joel.brie@unilim.fr (J.B.); eric.champion@unilim.fr (É.C.)

* Correspondence: amandine.magnaudeix@unilim.fr; Tel.: +33-5-87-50-24-43

† These authors contributed equally to this work.

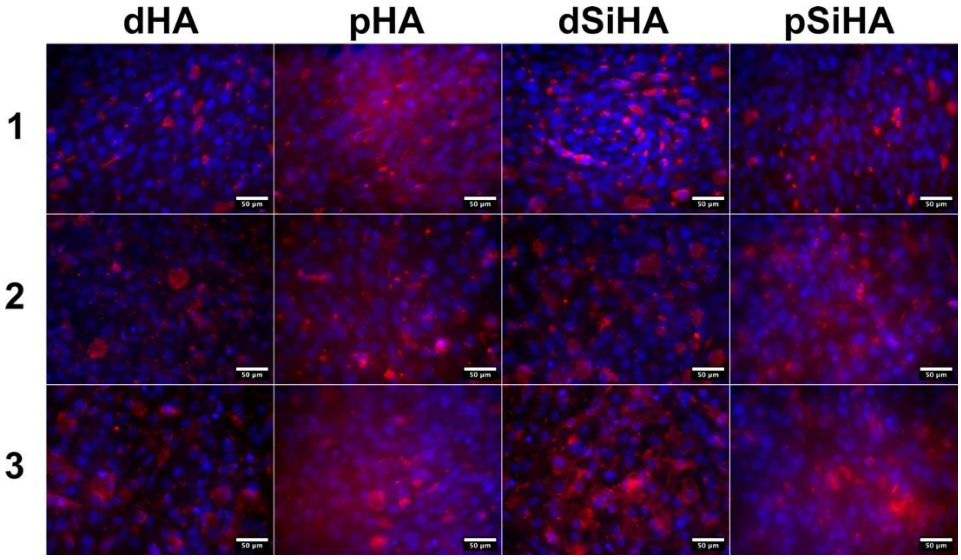

Figure S1. In situ expression of MMP-9 in C166 endothelial cells cultured on HA-based ceramic pellets after immunofluorescence staining. 1, 2 and 3 represent three independent experiments. Scale bar: 50 μm.

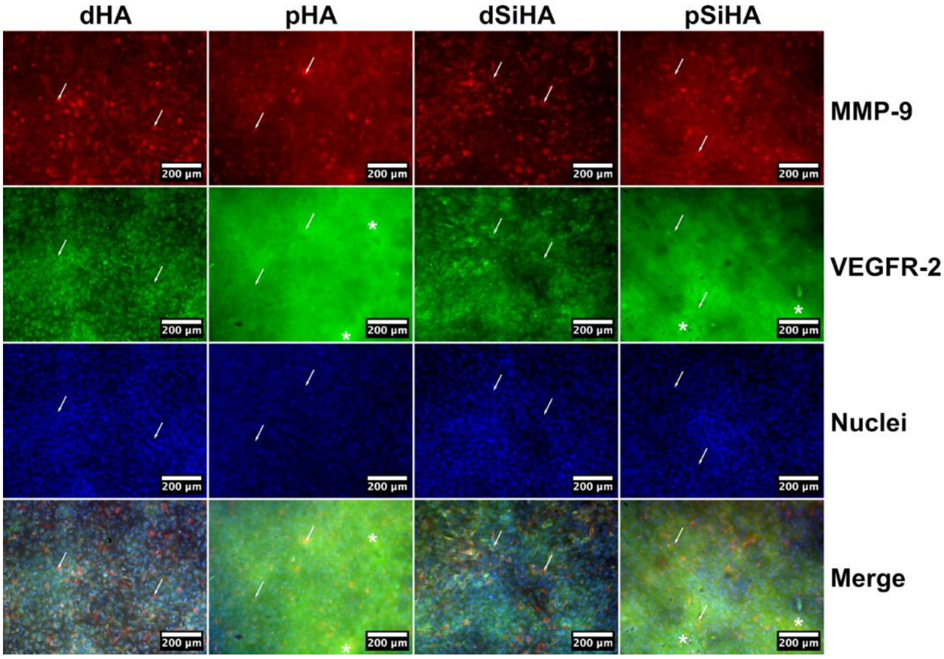

Figure S2. In situ expression of MMP-9 (red) and VEGFR-2 (green) as angiogenesis related-markers in C166 endothelial cells cultured on HA-based ceramic pellets after immunofluorescence staining. False colors were applied by post-processing of microscopy images. Nuclei were stained by Hoechst 33342 (blue). Scale bar: 200 µm.

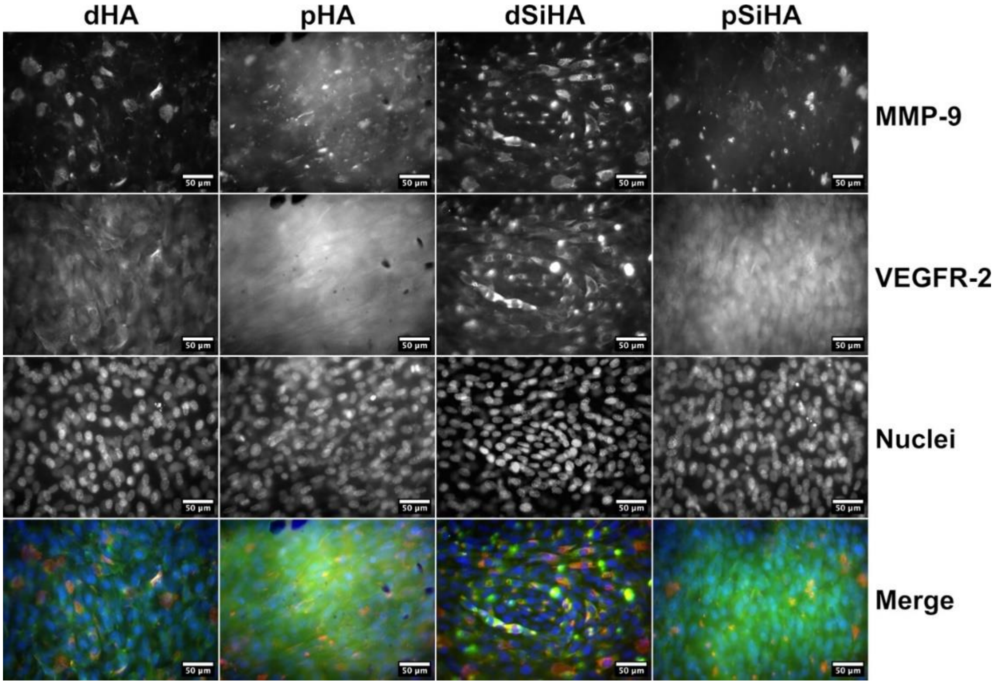

Figure S3. In situ expression of MMP-9 and VEGFR-2 as angiogenesis related-markers in C166 endothelial cells cultured on HA-based ceramic pellets after immunofluorescence staining. Scale bar: 50 µm.

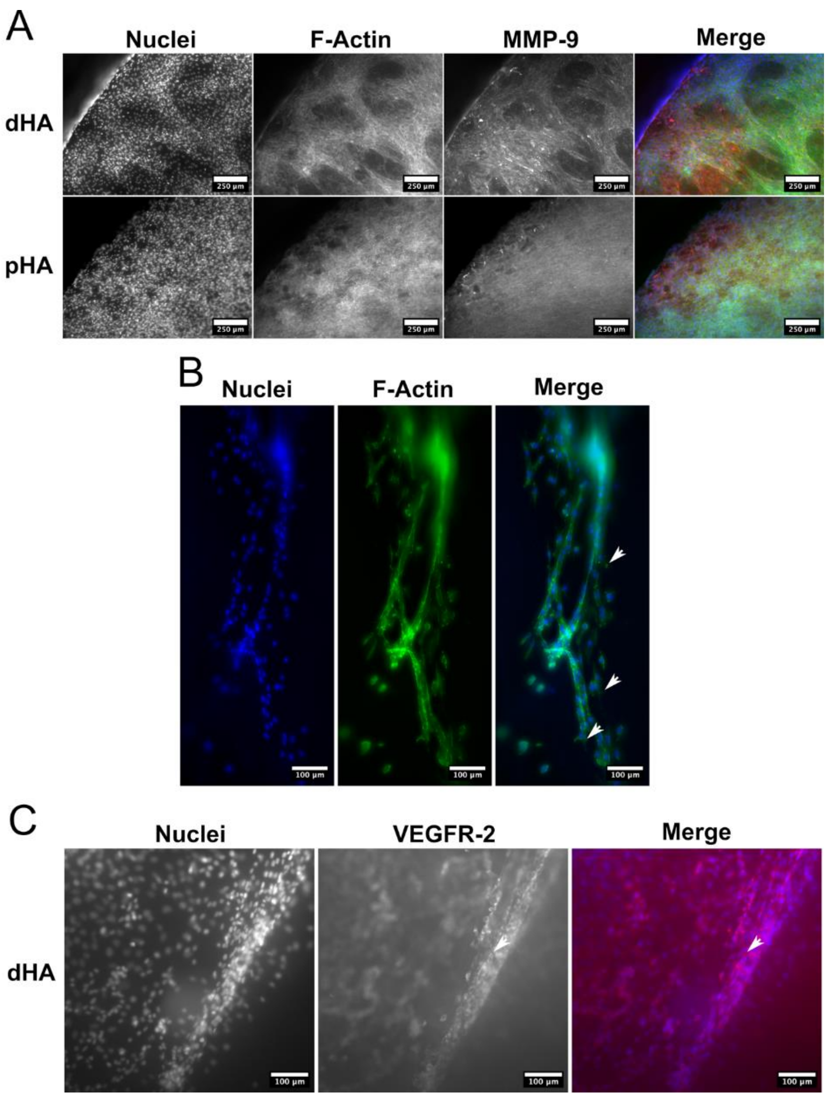

Figure S4. In situ visualization of C166 endothelial cells in fibrin gels (configuration 1). (**A**). Comparison of F-Actin (green) stained using phalloidin conjugated to DyLight 488 and MMP-9 (red) stained by immunofluorescence on dHA and pHA surface. Scale bar: 250 µm. (**B**). Tubule-like structures on the surface of dSiHA. Arrows point out cells with a tip-cell morphology. Scale bar: 100 µm. (**C**). VEGFR-2 immunostaining of endothelial cells on dHA surface. Scale bar: 100 µm.

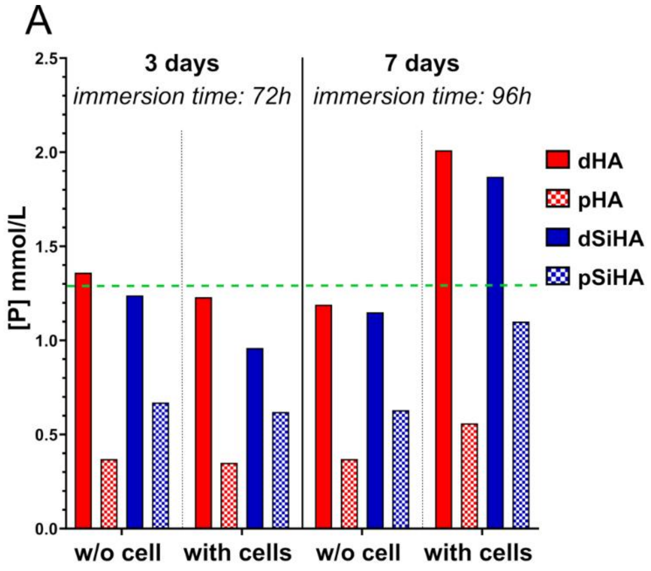

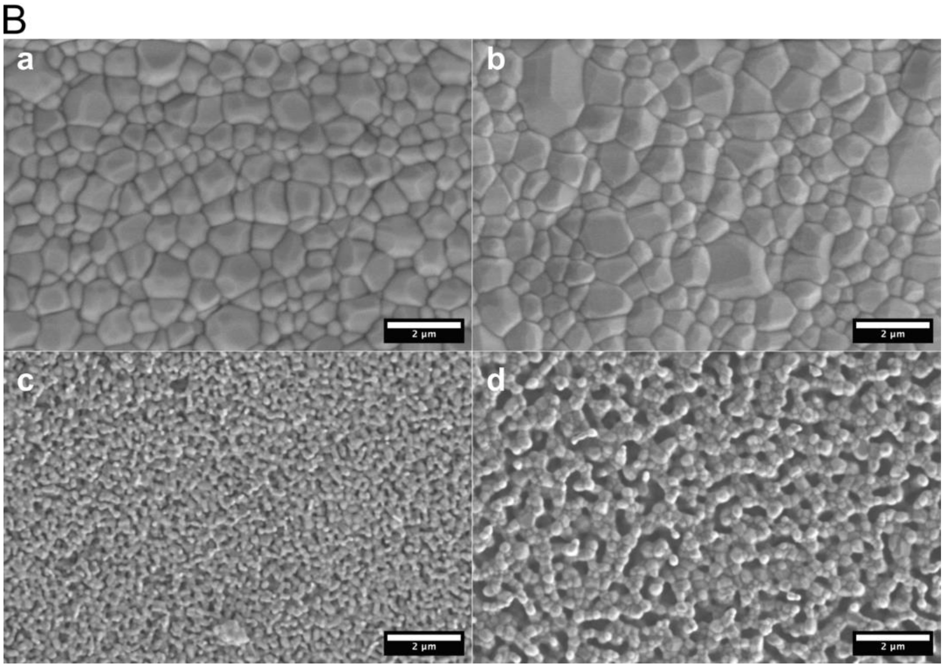

Figure S5. Ceramics pellets after immersion in complete culture medium for 7 days. (**A**). Phosphorus concentration in culture medium after immersion of ceramics with or without C166 cells, dosed by ICP-OES. The green line materializes the concentration of phosphorus measured in the complete culture medium alone. (**B**). Surface of ceramics in environmental scanning electron microscopy after immersion in complete culture medium for 7 days with a medium renewal after 3 days. Scale bar: 2 µm.

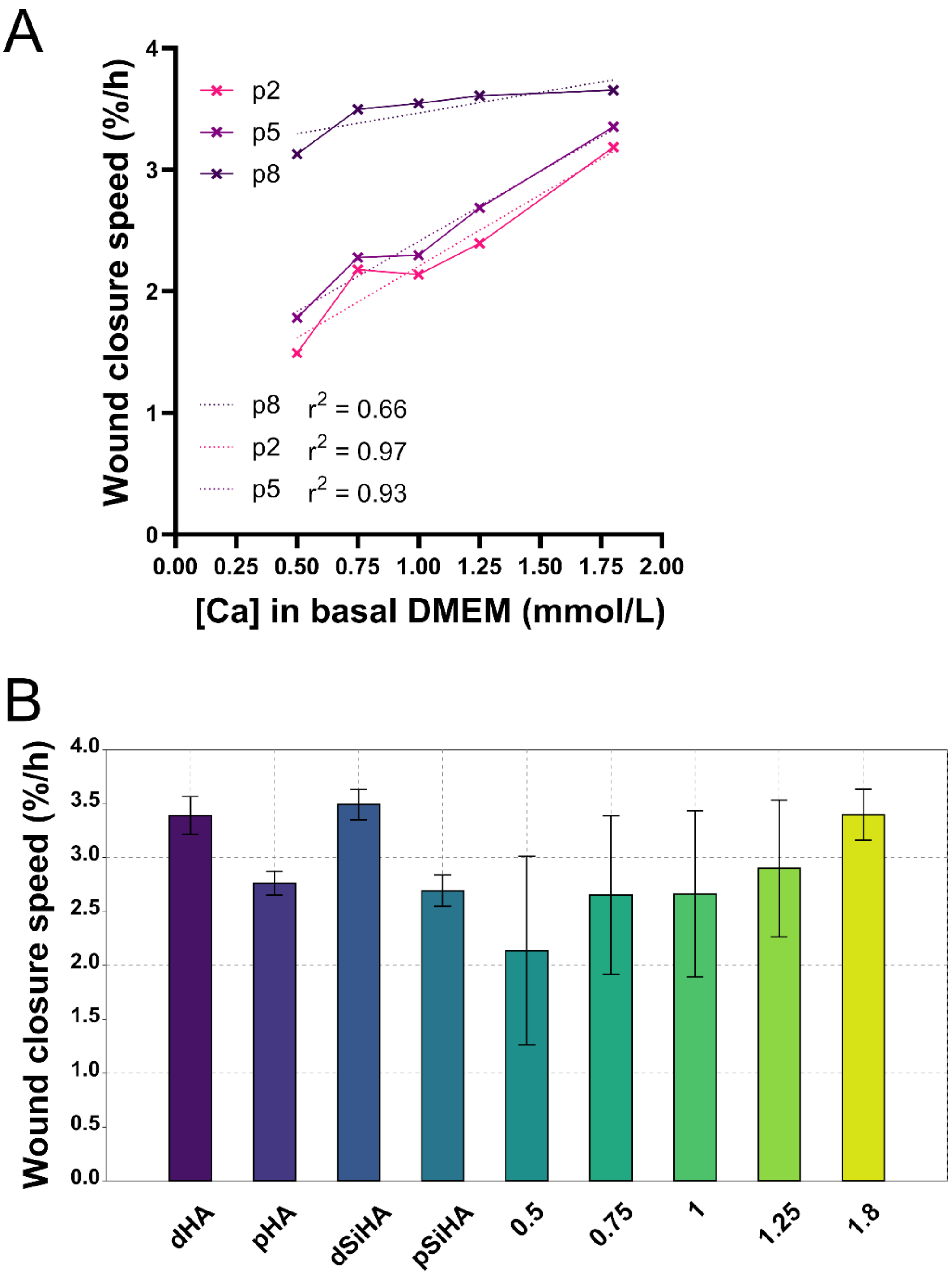

Figure S6. Wound healing assay performed for 48h on C166 cell layers cultured in complete culture medium with various concentration of calcium. (**A**). Wound closure speed according to the calcium concentration in the complete culture medium and the cell passage number. r2 is the correlation coefficient to qualify the linearity of the relationship between closure speed and calcium concentration. (**B**). Comparison of the wound closure speed, calculated during the first 24h at the surface of ceramics or in complete culture medium with different calcium concentrations given in mmol/L in basal DMEM (n= 3 independent experiments).
